# A Case of Dentin Dysplasia with Full Mouth Rehabilitation: A 3-year Longitudinal Study

**DOI:** 10.5005/jp-journals-10005-1248

**Published:** 2014-08-29

**Authors:** Suneet Khandelwal, Dheeraj Gupta, Lalit Likhyani

**Affiliations:** Reader, Department of Oral Pathology and Microbiology, Dr BR Ambedkar Dental College, Patna, Bihar, India; Reader, Department of Prosthodontics, Rajasthan Dental College Jaipur, Rajasthan, India; Assitant Professor, Department of Conservative Dentistry, Government Dental College, Jaipur, Rajasthan, India

**Keywords:** Dentin dysplasia, Opalescent dentin, Pulpless teeth, Radicular dentin dysplasia, Rootless teeth, Thistle tube teeth

## Abstract

Dentin dysplasia, a rare hereditary disorder of dentin formation, is characterized by normal enamel but atypical dentin formation along with abnormal pulpal morphology. It is inherited as an autosomal dominant trait. It has been divided into two clinical entities: type I (radicular) and type II (coronal). Early diagnosis and initiation of effective regular dental treatments may help the patients with this condition to delay or prevent the loss of the entire dentition and help them in cope up with edentulous state in early ages. The condition undoubtedly has a negative impact on the physical and psychological well-being of the affected individual. Numerous factors have to be considered during the prosthetic rehabilitation of patients with dentin dysplasia. Treatment protocol varies according to clinical case. Although literature reports suggest general guidelines for treatment planning, the present case report describes a full mouth rehabilitation of an 8-year-old female patient with dentin dysplasia.

**How to cite this article:** Khandelwal S, Gupta D, Likhyani L. A Case of Dentin Dysplasia with Full Mouth Rehabilitation: A 3-year Longitudinal Study. Int J Clin Pediatr Dent 2014;7(2): 119-124.

## INTRODUCTION

Dentin dysplasia (DD) is an autosomal dominant hereditary disturbance in dentin formation, which may present with either mobile teeth or pain associated with spontaneous dental abscesses or cysts. This rare anomaly of unknown etiology, affects approximately one patient in every 100,000.^1^ Balchsmiede in 1920 first reported this condition as ‘root less teeth’ which was later described as ‘dentinal dysplasia’ by Rushton in 1939. The disturbance of dentin development was recognized, such as highly atypical dentin, accompanied with obliteration of the pulp and defective root formations. Stafine and Gibilisco later reported three cases with similar radiographic findings but there were no microscopic descriptions.^2^

Carrol O et al proposed a subclassification based on the radiographic findings. They proposed two basic types. Dentin dysplasia type I is characterized by the presence of primary and permanent teeth with normal appearance of the crown but no or only rudimentary root development, incomplete or total obliteration of the pulp chamber and periapical radiolucent areas or cysts. DD-I was classified into four subtypes: Ia, Ib, Ic and Id. Type Ia is characterized by a complete obliteration of the pulp and usually little or no root development. Subtype Ib has a horizontal, crescent shaped, radiolucent line, separating normal coronal dentin from abnormal radicular dentin and short, conical and rudimentary roots. Type Ic affected teeth shows two crescent-shaped horizontal radiolucent lines with their concavities toward each other at the cement-enamel junction. The roots are one half the normal lengths. Normal root formation, which sometimes may be bulbous in the coronal third, is seen in type Id. A stone may be found within the pulpal canal. In this least severe type of DD, normal root formation occurs and the pulp chamber is usually not obliterated. In these cases, the pulp around the stones is healthy. In other cases, the denticle is continuous with dentinal walls.

Unlike the first variant, DD type II is characterized by primary teeth with complete pulpal obliteration and brown or amber bluish coloration similar to that seen in hereditary opalescent dentin. The permanent teeth have a normal appearance or a slight amber coloration; the roots are normal in size and shape with a thistle-tube shaped pulp chamber due to sudden constriction of the chamber with pulp stones.^3^

The sequelae of dentin dysplasia are difficult to manage and provide a challenge for the dentist concerning restorative and endodontic treatment but also prosthetic treatment after loss of teeth.^4^ This report highlights the multidisciplinary treatment approach of a female child suffering from DD-I facing almost complete edentulous state.

## CASE REPORT

An 8-year-old girl reported with complaints of pain in gums and difficulty in eating since 1 year. Her most of the teeth fell down within a short period of eruption, as reported by her mother. There were no previous cases of this disturbance in the familial history and was nothing significant in her medical and personal history.

**Figs 1A to C:**
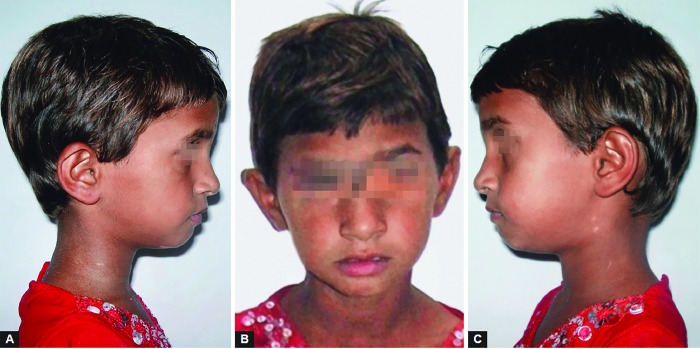
Extraoral left lateral, front and right lateral profile of the patient’s face

**Figs 2A and B:**
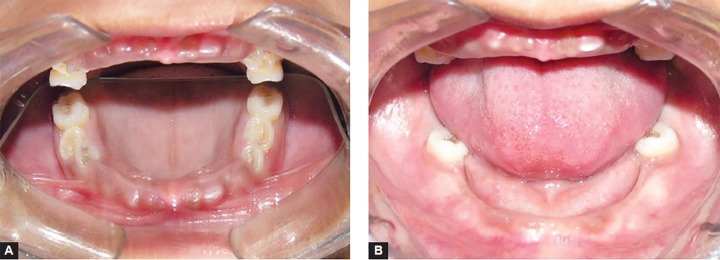
Intraoral view of the maxillary arch and mandibular arch before treatment

General physical and intellectual examination revealed the normal findings except her discolored hair, which was similar to her mother (Figs 1A to C). The intraoral clinical examination revealed only eight teeth (4 permanent first molars and 4 maxillary primary molars). There was second grade mobility in 64, 26 and 36 without the history of trauma and infection. Teeth were smaller in shape and size, normal to opalescent in color, with severe attrition and cavitation as well. Anterior area in maxillary region showed the evidence of eruption of permanent teeth. Occlusal contact relationship was present only at first permanent molar region. Alveolar process in the region of missing teeth remains undeveloped. There were no abnormalities detected in intraoral soft tissues (Figs 2A and B).

**Fig. 3:**
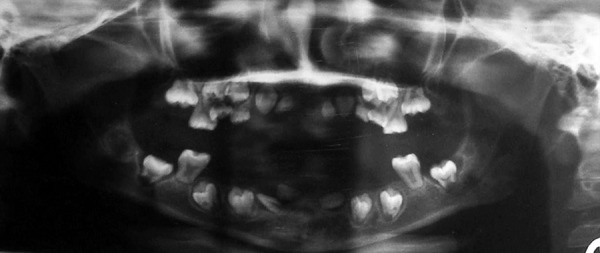
Orthopantomogram shows typical appearance of teeth affected with DD-I, with underdeveloped roots, narrow pulp chambers and periapical radiolucencies

Orthopantomogram (OPG) was done to evaluate the status of erupted and developing dentition. Orthopantomogram (Fig. 3) confirmed the evidence of all permanent successors as per normal chronologic developmental order except all central incisors and left lower lateral incisor which were missing. The panoramic radiographs showed features characteristic of dentin dysplasia type I with normal appearance of the crown but underdeveloped roots of all teeth, periapical radio-lucencies and narrow and half-moon shaped pulpal space because of thickened dentin. Blood investigations (serum calcium – 8.8 mg%, serum phosphorous – 4.1 mg%, PTH assay 28.50 pg/ml, alkaline phosphatase – 465 IU/l) were in normal range.

Based on the clinical examination and investigations, final diagnosis of dentin dysplasia type I was made.

**Figs 4A and B:**
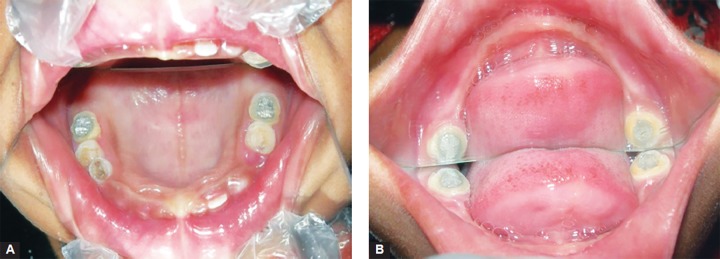
Teeth of maxillary and mandibular arch after restoration at 1 year follow-up

**Fig. 5:**
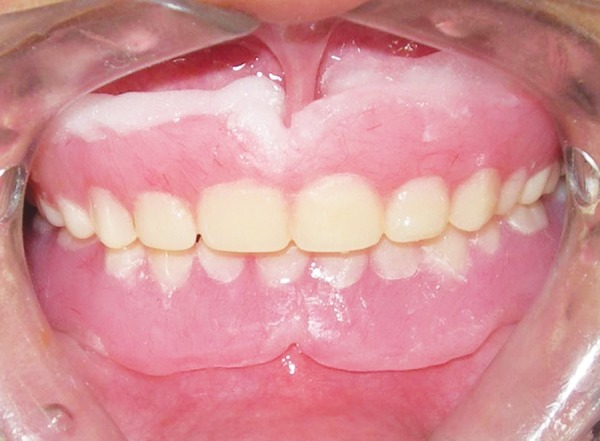
Application of soft liner over the denture at 1 year follow-up

**Figs 6A and B:**
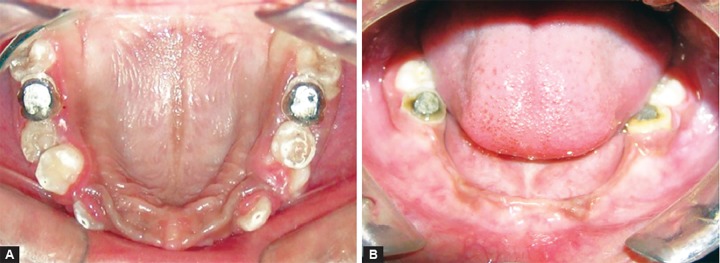
Intraoral view of the maxillary and mandibular arch at 3 years follow-up

**Figs 7A to C:**
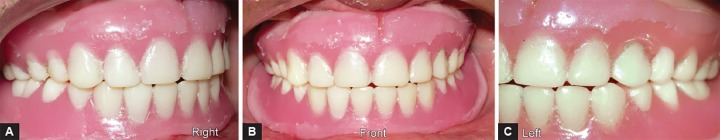
Intraoral view of the denture at 3 years follow-up

**Fig. 8:**
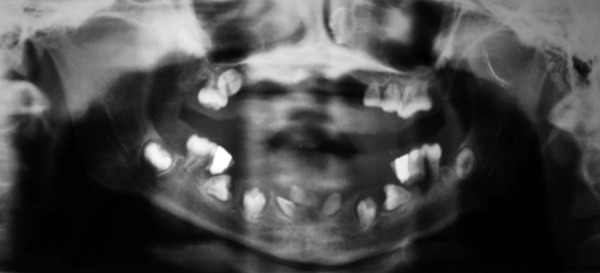
Orthopantomogram shows status of dentition affected by DD-I after 3 years

**Figs 9A and B:**
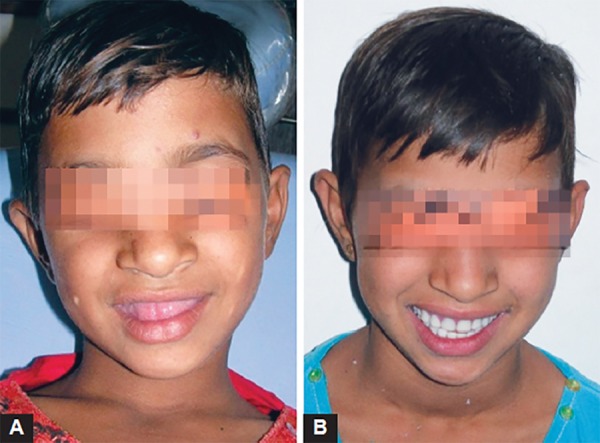
Smile of the patient before and after 3 years of the treatment showing ample of confidence and attitude changes

Patient was treated with using multidisciplinary approach. Based on pedodontist’s and endodontist’s view, extraction of 64 and restoration of all the decayed teeth (54, 55, 16, 65, 26, 36 and 46) were planned and performed (Figs 4A and B). Following this, a complete over denture with soft liner was made under the guidance of prosthodontist to restore the normal esthetics and masticatory function (Fig. 5). Patient was advised to come for regular follow-up visits to make necessary alterations in the denture following eruption of permanent teeth. Both upper permanent canine and right first premolar were got extracted in due course of time. Continuing follow-up visits and few adjustments after 2.5 years, a new set of denture was made considering growth and development in dentition and otherwise (Figs 6 and 7). Altogether four teeth were removed till date (Fig. 8) and sent for histopathological examination. A considerable change in the patient attitude and confidence level was noted (Figs 9A and B).

**Fig. 10:**
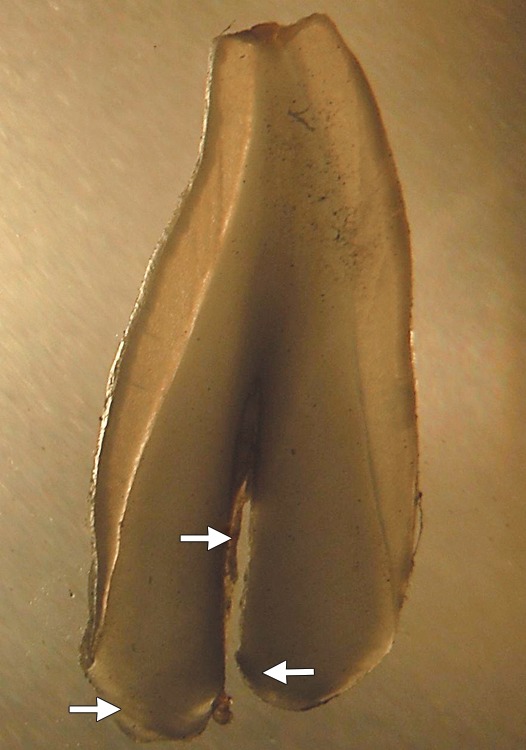
Ground section of exfoliated tooth examined under stereomicroscope shows narrow pulpal space and abnormal radicular dentin

Ground section of extracted tooth under stereomicroscope revealed thickened dentin and narrow pulpal space (Fig. 10). As roots of the extracted teeth were of very small size though the abnormality in radicular dentin was noted. These features are consistent with those of DD type I, confirming the diagnosis based on the clinical and radiographic features.

It is anticipated that unerupted permanent teeth may undergo premature removal as erupt into the oral cavity. The possibility of endosseous implants is being explored for when the patient reaches her late teens and growth is complete.

## DISCUSSION

Little is known about etiology of DD in healthy individuals. Several factors possible causes have been implicated, but the exact mechanism of the defect is yet to be determined. Dentin contains collagen type I and II, the abnormality of which is presented as DD of different forms and severity. DD-I is an inherited disorder characterized by atypical development of the dentin. This disorder is also known as radicular dentin dysplasia as the underdeveloped, abnormal pulp tissue is predominately in the roots of the teeth. The teeth either lack pulp chambers or have crescent shaped pulp chambers with short or abnormally shaped roots. This abnormal root morphology is thought to be secondary to the abnormal differentiation and/or function of the odontoblasts. The condition may affect both the dentition and, since the roots are abnormally short, premature loss of teeth is usually seen while, the color of the teeth is usually remains normal. DD-II is characterized by primary teeth with complete obliteration of the pulp. The teeth shows brown or amber bluish coloration similar to hereditary opalescent dentin. The permanent teeth, however, have a normal appearance or a slight amber coloration, the roots are normal with a thistle-tube shaped pulp chamber with pulp stones.^3,5,6^

Different theories have been postulated to explain the pathogenesis of the disease. The first theory states that dental papilla is responsible to the extent with multiple foci of degeneration of the same that calcify and, during the healing period, obliterate the pulp space. The second states that the early invagination of the epithelium in the embryonic process subsequently generates multiple foci of dentin formation. The third states the alteration in the normal interaction that exists between the layer of the odontoblasts and ameloblasts to be responsible for the abnormal dentin formation.^7^

Mutations in two genes, MSX1 and DSPP, have also been thought of in the disease pathogenesis in a recent study. The DSPP gene was considered within the study as candidate gene for the etiology of the dentin dysplasia type I, due to the description in the literature of mutations in exon 2 of the DSPP gene in patients with dentinogenesis imperfecta. The mapping on chromosome 4q21.3 of the dentin dysplasia type II region in which the DSPP gene encodes also aided in recognizing this gene as the candidate gene.^7,8^

In general, the diagnostic criteria rests solely on clinical grounds; however, as the genetic mutations underlying these conditions are now being recognized, molecular genetic diagnosis may serve as an useful adjunct to clinical evaluation, particularly when there is a doubtful diagnosis. Vitamin D-dependent rickets and vitamin D-resistant rickets have clinical and radiographic features of dentinogenesis imperfecta-I and DD. Chemotherapy or irradiation of the jaws during the period of root development leads to arrested development and can give a radiographic appearance similar to DD-I.^9^ Variety of congenital syndromes, including familial calcinosis and the brachioskeletogenital syndrome and Ehlers Danlos syndrome, is a systemic condition of genetic disorders of collagen in which patients reveal skin abnormality and joint hypermobility have been reported in association with DD.^5,6^

Life expectancy of dentition is minimal, although various treatment strategies, including conventional endodontic therapy, periapical curettage or preventive regimen, have been proposed to maintain the teeth as long as possible. Early exfoliation of the teeth and associated maxillomandibular atrophy is seen in DD-I.^7^ Maxillomandibular atrophy is a consequence of no or partial root development and early tooth loss. Successful oral rehabilitation with complete denture after extraction of all teeth and curettage of cysts has been described.^10^

The treatment plan aims to remove the infection or pain, restores esthetics and protects posterior teeth from wear. Treatment may vary given the age of the patient, severity of the problem and the presenting chief complaint. As in the primary dentition, one of the options is overdenture in permanent too. Those with DD-I have mobile teeth due to very short roots and, as a result, there is early exfoliation of teeth in the primary and permanent dentition.^9,10^ Until growth is complete, the treatment of choice for the replacement of missing teeth is dentures. Ridge augmentation followed by dental implants may be considered at about 18 years of age, when growth is completed.

Exposed dentin is more susceptible to caries than enamel. Regular dental checkups and caries prevention by means of oral hygiene instructions, diet counseling and appropriate fluoride application is essential. Early diagnosis and regular dental check-ups however do not aid in preventing premature exfoliation.^9^

## CONCLUSION

The outcome of a diagnosis of DD largely depends upon the age at which the condition is being diagnosed and the rapid treatment protocol. Where diagnosis occurs early in the life of the patient and treatment follows the above recommendations, good esthetics and function can be obtained thereby minimizing nutritional deficits and psychosocial distress.
